# Vaccininum and Variolinum

**Published:** 1893-07

**Authors:** John Hall

**Affiliations:** Victoria, B. C.


### VACCININUM AND VARIOLINUM.

*To the Editor of the Homœopathic Physician.*

DEAR SIR:—I have looked in vain among the pages of your journal for some reply to the excellent paper of Dr. Stuart Close on "Vaccination," in the number for November, 1892, p. 468, wherein he quotes Dr. Hering as considering both *vaccine and variolin as identical.* I am sorry to differ from so high an authority as Dr. Hering, for no one can think more of him than myself, but the subject he has handled in the *Guiding Symptoms* calls for a reply, owing to its grave importance. I had commenced a paper on this matter immediately on reading Dr. Close's article, but feeling sure that younger and better men would do it more justice, had refrained from continuing; however, was surprised that no one attempted to deal with this subject.

Let me say, then, that the matter which Dr. Jenner used was derived from *various sources,* as can be learned *from his own statements,* and though the Parliament of that time granted him £30,000 for his discovery, it has been *abundantly proved by subsequent investigation* that this protection to our species by vaccination is by no means certain—that the halo which surrounded us when submitting to this rite has disappeared. Even the members, or many of the most distinguished in Parliament, have said on the floor of the house that the bill of *compulsory* vaccination had been *foisted* on *them* and passed by *false statement,* against which has arisen a society which seeks its abolition. A society which numbers among its members some of the first names in Britain, and has spread nearly all over Europe, fondly expecting that the obnoxious and unconstitutional law will shortly be expunged from the statutes.

We have had here an epidemic of small-pox, against which some of the most foolish decrees have been instituted, resulting in frightening the people so greatly that trade and commerce have been sadly paralyzed. To be sure, we are called upon to "stamp it out," as it is termed, and other towns expect this to be done, whether by vaccination or re-vaccination, quarantine, etc., means which have no effect whatever in controlling the disease, but which is easily and rapidly done by a *very high potency* of Variolin, *not vaccine,* the same remedy in *one dose* effectively preserving those who take it from ever suffering from the malady in their lives, provided they be of fairly good habits.

But I begun this paper to show that the two nosodes were not identical—the one fully curing and preserving, the other acting in a very uncertain manner, as we may see in the very bad cases which have occurred in those who have been vaccinated recently, several of whom have died.

But I must draw this subject to a close, merely remarking that the divergence of the two nosodes can be best learned from Dr. Jenner's own words, and that when the wave of small-pox epidemic passes over, the disease leaves us, and not till then.

VICTORIA, B. C., January 3d, 1893.

JOHN HALL.

